# The Occupational Narratives of Older Adults Participating in Music-Based Occupations

**DOI:** 10.1093/geroni/igab046.1988

**Published:** 2021-12-17

**Authors:** Penelope Moyers Cleveland, Kassidy Beckstein, Allie Gartner, Lexy Hay, Mackenzie King, Sammy McLeish, Courtney Romatz

**Affiliations:** University of Indianapolis, Indianapolis, Indiana, United States

## Abstract

The purpose of this study was to implement an occupational therapy intervention that could be used for telehealth services with an emphasis on participants learning ways to independently choose and sustain engagement in meaningful music activities, known as occupations. The researchers’ aim was to examine how music occupation interventions lower risks of occupational deprivation (i.e., prolonged restriction from participation in necessary or meaningful activities) that could occur due to the COVID-19 pandemic. Eight adults participated who were 65 years or older, lived in the community, and enjoyed music. The researchers used narrative qualitative methodology to analyze pre- and post-intervention focus group data. The participants completed seven intervention sessions designed to increase and sustain music engagement outside of the sessions. The pre-intervention focus group data resulted in an occupational pattern analysis and a single occupational narrative. Triangulation of data post-intervention included the two focus groups and their pattern analyses and narratives, field notes from each intervention session, and documents produced through group completion. The final analysis produced an occupational change pattern analysis and narrative. The focus of the change narrative was on the participant’s management or prevention of occupational deprivation. The researchers identified several common themes involving change in routines and habits to include regular engagement in meaningful music activities, skills for using occupational participation as an important method of coping with COVID-19, and developing new technological skills to access music to replace in-person participation of attending live concerts and shows when deemed unsafe because of potential for virus transmission.

